# Aiming to improve the quality of primary mental health care: developing an intervention for underserved communities

**DOI:** 10.1186/1471-2296-15-68

**Published:** 2014-04-16

**Authors:** Carolyn Chew-Graham, Heather Burroughs, Derek Hibbert, Linda Gask, Susan Beatty, Katja Gravenhorst, Waquas Waheed, Marija Kovandžić, Mark Gabbay, Chris Dowrick

**Affiliations:** 1 Research Institute for Primary Care and Health Sciences, Keele University, Keele, UK; 2 Institute of Population Health, University of Manchester, Manchester, UK; 3 Institute of Psychology Health and Society, University of Liverpool, Liverpool, UK

**Keywords:** Mental health, Underserved populations, Primary care

## Abstract

**Background:**

The purpose of the study was to improve the quality of primary mental healthcare in underserved communities through involvement with the wider primary care team members and local community agencies.

**Methods:**

We developed training intended for all GP practice staff which included elements of knowledge transfer, systems review and active linking. Seven GP Practices in four localities (North West England, UK) took part in the training. Qualitative evaluation was conducted using thirteen semi-structured interviews and two focus groups in six of the participating practices; analysis used principles of Framework Analysis.

**Results:**

Staff who had engaged with the training programme reported increased awareness, recognition and respect for the needs of patients from under-served communities. We received reports of changes in style and content of interactions, particularly amongst receptionists, and evidence of system change. In addition, the training program increased awareness of – and encouraged signposting to - community agencies within the practice locality.

**Conclusions:**

This study demonstrates how engaging with practices and delivering training in a changing health care system might best be attempted. The importance of engaging with community agencies is clear, as is the use of the AMP model as a template for further research.

## Background

The management of people with common mental health problems represents a significant part of general practitioners’ (GPs) daily work [1,2]. Many people with high levels of mental distress are disadvantaged either because care is not available to them at the right place and time, or because when they do access care their interaction with care-givers deters help-seeking or diverts it into forms that do not address their needs [3].

It is known that simply offering training to GPs, even if this includes work on skills as well as knowledge, does not lead to improvement in outcomes for patients with mental health problems [4-7]. However, Sikorski et al. [8] suggest that although training by itself does not improve care or patient outcomes, if combined with additional guidelines implementation, results may be promising for newly diagnosed patients with depression. Wensing et al. [9] report that multifaceted interventions are more effective than single interventions in changing practitioner behaviour. They found that using a combination of basic information transfer with learning through social influence was more effective, and that the combination of three or four different approaches was more successful than a single intervention.

Doctor-dependent cultures can stifle improvement and innovation [10,11] and the focus has moved to the practice team. Improvement of primary care quality and organizational change are both important influences on patient outcome [12] but any initiative needs to be seen as relevant to the practice’s everyday work, and flexible enough to respond to the challenges posed by the needs of an individual practice [13] New integrated models of care are needed for patients with complex and long-term care needs [14], and the focus needs to move from simply offering training to individual practitioner groups, to multidisciplinary training and organizational change. However, non-GP members of the practice team who are not used to being involved in planning, assessing improvement or participating in training can be anxious about expectations to do so [15].

Previous studies have identified that efforts to change organizational and professional practice are best preceded by the effort to understand what is already happening [16]. Interventions also need to be tested within context, and the contextual forces (the people, the community, the resources, etc.) need to be considered along with the intervention to determine their contribution to the outcome [17].

Quality improvement in primary care appears likely to benefit from considering not only the roles of the wider primary care team but also the needs and perspectives of the local community which it seeks to serve. Community engagement efforts “enhance the public trust in clinical and translational research” through “long-term relationships with community-based groups” [18] In the United States there is a call for the creation of ‘Communities of Solution’ [19] where physical and mental health are considered intrinsically linked to housing, poverty and work. The concept of ‘space of access’ developed within our team, stresses the importance of “dynamics of resources beyond the ‘medical zone’ of care” and a need for reshaping primary mental health care by “de-centering and re-connecting the role of medical professionals” [20]. Tailoring education to fit with the audience also means developing and delivering it locally, based on identified needs and making it relevant to the needs of the local community [21]. In addition to improved training and retention of primary care physicians, a network of care that extends into the community is considered necessary, with communities identifying their own health-care needs and thus facilitating local improvement.

**Figure 1:**
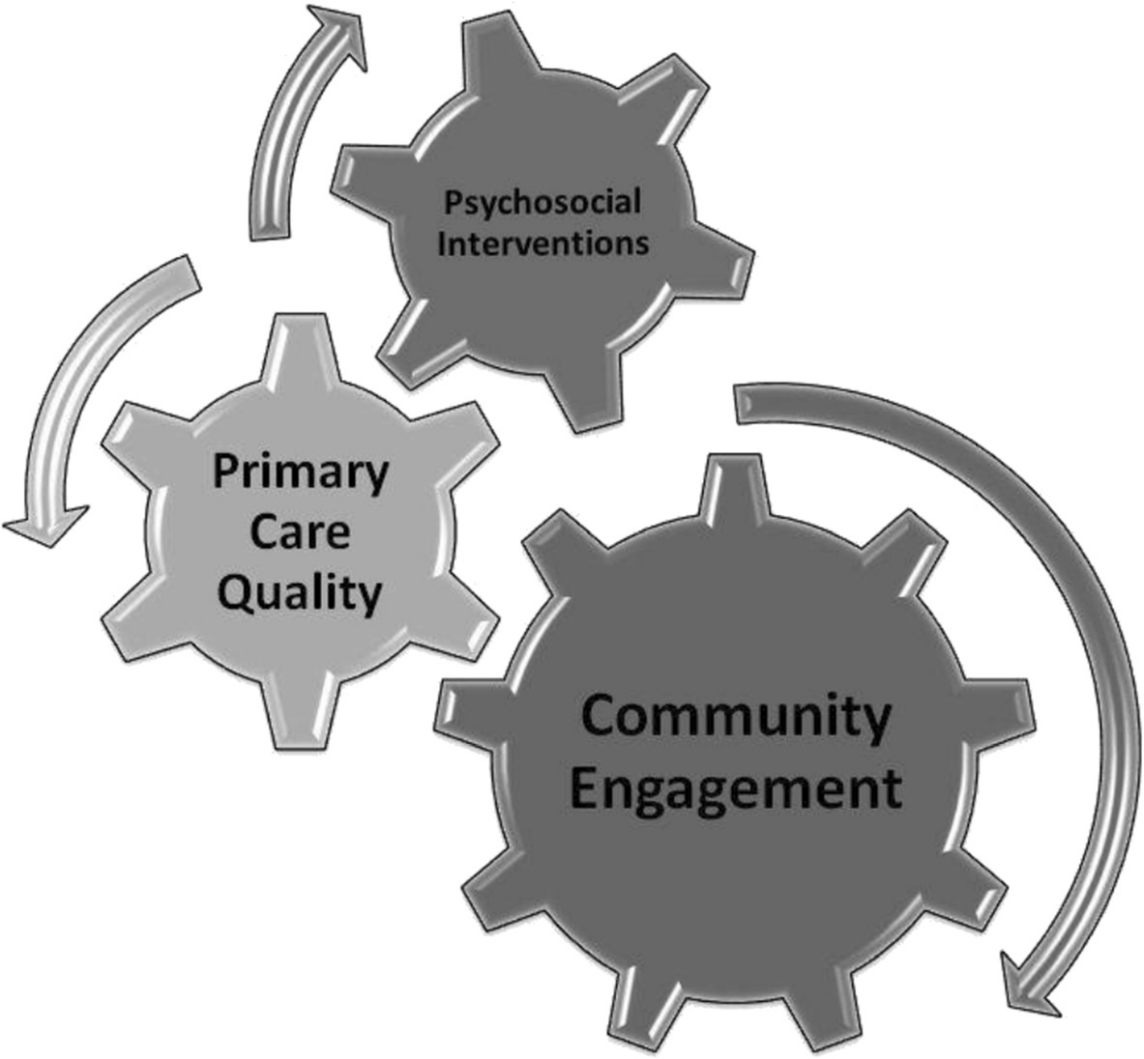
**The AMP model: a multi-faceted model of care **[24]**[uploaded as an additional file].**

The overall aim of the AMP (Access to Mental health in Primary care) Programme [22] was to increase access to high quality primary care mental health services for people from under-served groups. The underlying rationale of the AMP model is the need to promote primary care services that recognize and accommodate the various ways that under-served users, and their communities, frame or understand common mental health problems. This puts the initiative broadly in line with ideas of ‘patient-centred’ [23] and ‘culturally-responsive’ [24] services. We designed a new multi-faceted model [25] (see Figure 1).

The model aimed to improve access to primary care mental health for people from under-served communities. The three core components of the AMP model [24] are explained below.

• **Community engagement.** Community engagement provides the opportunity to improve wellbeing and access for individuals, and the resourcefulness of a system as a whole. It aims to meet local needs and presents an opportunity for reclamation of identity and agency, and includes the option of improved access to sensitized primary care services.

• **Primary care quality.** To improve the quality of patient experience when members of hard-to reach and underserved groups access primary care, primary care teams need to increase their competence in understanding and responding to the differing ways in which members of under-served groups present suffering, and in encouraging them to access relevant services.

• **Psychosocial interventions.** There is evidence for the effectiveness of psychosocial interventions for many under-served groups. Interventions must be tailored to meet the personal and communal needs of those who may benefit from them.

This paper presents the development and evaluation of the Primary Care Quality component, and explores whether quality improvement in primary care is enhanced by the involvement of the wider primary care team and engagement with the local community.

## Methods

### Development of AMP training*plus*

We built on the earlier work by AMP programme members [26-28] to develop an intervention to improve Primary Care Quality, which we called AMP training*plus*.

Evidence contributing to the development of AMP training*plus:*

1) Systematic review of access studies,

2) Meta-synthesis of data on patient perspectives,

3) Dialogues with local stakeholders,

4) Review of grey literature from statutory and voluntary service providers,

5) Secondary analysis of patient transcripts from previous qualitative studies,

6) Primary data from interviews with service users and carers.

The results of the 6-stage evidence gathering process suggested that the best approach would be a quality improvement intervention which would offer a flexible training package for primary care teams tailored to fit with local needs and priorities. To ensure this tailoring, 200 hours of ethnographic observation was carried out in seven practices [29]. This enabled us to get to know members of the practices, learn how the practice worked on a day-to-day basis, establish trust and generate the feeling that we were entering into a partnership with the practices rather than making them the subjects of research. With this ethnographical knowledge we were able to offer practical support to review and address local organizational barriers to access e.g. appointment booking, consultation times and interpreter services. The intervention was intended to support practices to develop and utilize links to a wide range of internal and external resources, including tailored community-based psycho-social interventions developed as part of the AMP intervention.

The resulting quality improvement intervention (AMP training*plus*) had three interlinked strands:

• *Knowledge transfer*: including a training component of up to six sessions, selected by practice members from a menu of subject options. Our aim was to foster a space within which team members could reflect on practice and learn from colleagues.

• *Systems review*: we offered practices intensive observation (up to one week) to identify organisational and structural features that may impede or promote access by under-served groups. This was situated in reception areas focusing on appointment booking systems.

• *Active linking*: we offered to raise awareness of other relevant local organisations and resources, which had been mapped and logged by the AMP team.

The knowledge transfer (KT) component was based on Grol’s model [30] which emphasizes the need to combine expertise from specialists with co-production of ‘new’ knowledge within the practice. AMP training*plus* was developed for all practice staff, both those who had clinical and administrative contact with service users. We intended it to be practice-centered and responsive to the needs of the practice, and to include topics such as consultation skills (detection of mental health problems, negotiating diagnoses, initial management, psychosocial skills, behavioral activation) and topics of broader relevance such as cultural competence, exploration of personal attitudes and values, and working with interpreter services. The particular emphasis would depend on the local priorities determined by practices and primary care teams.

We identified the following outcomes that we hoped to achieve:

• Increased staff awareness, recognition and respect for diversity

• Change in consultation or encounter style and content, including communication, listening and negotiation, use of evidence-base, models of illness, use of interpreters, use of motivational techniques

• Change in consultation or encounter outcomes, including referral, signposting, prescribing and satisfaction

• Change in processes and systems, for example, booking systems, use of interpreters, and referral to psychosocial interventions and social prescribing.

### Delivery of AMP training*plus*

AMP training*plus* was one of the three components in the AMP model. Working with our Primary Care Trust (PCT) partners, we identified eight general practices in 4 deprived localities which were invited to take part in the AMP programme [31]. We also attempted to recruit practices to become involved in the AMP programme as “control” practices, which would not participate in training, but would agree to collect data on referrals for people with mental health services. Our aim was also to recruit two practices in each of the four localities selected to participate as intervention sites, which were to receive the training. The practices were sent letters of invitation to named senior GP partners and to practice managers along with written information explaining the purpose and nature of AMP training*plus*. Members of the research team then attempted to contact the named GP (and other GPs in the practice) and the practice manager to ascertain interest in the study.

At a later stage we also approached the two practices in each locality that had been randomised to act as control sites and not receive the training. We sought to meet with members of the practice team to introduce the programme of work and the broader aims of the AMP programme. In this meeting we aimed to negotiate the possibility of GPs and practice nurses using agreed computer codes to record the management of people with identified mental health problems.

When a practice expressed an interest in the programme, we arranged one or two pre-meetings to introduce the AMP programme and researchers, and answer questions about involvement in the study.

Topics covered in the initial meeting included summarising the results from phase 1 of the AMP programme; introducing community mapping (and community engagement for those localities where this was part of the intervention); negotiating conducting ethnographic observations in the practice. At the preliminary meeting(s) we also negotiated the initial training session, at which we would present the AMP model and the results of the community mapping and practice ethnographic observations, and explore local applicability and the practice’s learning needs. Practices were advised that the AMP team would facilitate practical arrangements for the first and any subsequent training sessions (rooms, equipment, refreshments, etc.). We offered financial remuneration to cover the time that staff spent participating in AMP training and activities, which could include payment for locum cover or reimbursement for the use of the out-of-hours service.

The initial training session was the same for all practices and covered the following areas: aims of the AMP programme, feedback of results of earlier phases of the AMP research programme, report of community mapping, report of ethnographic study of practice0 if that had been complete prior to this initial session (which was the case in all practices except one), discussion of learning needs of individuals within the practice and the group itself, and discussion of the potential for further work or training based on issues arising from the meeting.

After the initial training session we provided written feedback to each practice, summarising the issues discussed and suggesting possible areas where the AMP team could contribute in supporting the practice to meet some of the learning needs generated in the session. We opened discussion with the practice contact about content, format, invited speakers and staff attending for future sessions. We offered the practice a very broad menu of training options, and assured them that whatever training they wanted, we would try to arrange it. In fact we were able to fulfil all of the practice’s requests.

In the control practices we aimed to negotiate the possibility of GPs and practice nurses using agreed computer codes to record the management of people with identified mental health problems. A researcher visited non-intervention practices to discuss and gain agreement for the clinical staff to use the same codes in consultations with people with mental health problems. This data is not presented in this paper, as it became clear (at practice visits by researchers) that practices were not consistently using these codes.

### Subsequent training sessions

The structure and content of subsequent training sessions differed between practices, depending on individual practice wants, needs and negotiated aims. Table 1 summarises the topics chosen and covered in these sessions:

**Table 1:** **Topics covered in AMP training*plus *training sessions**

**Knowledge transfer (including skill development)**	**Systems review**	**Active linking**
Mental health in older people	Access and triage	Link with AMP community engagement sites
Patient presentations	The patient journey	Availability of local community groups and resources
Culture and mental health	The appointment system	How to work with community groups
Working with interpreters	Working with interpreters	AMP wellbeing Intervention
Symptom recognition by non-medical staff	Communication within the practice	Referring on (eg drug teams; social services)
Consultation skills for clinicians		
Communication skills for non-clinicians		
Managing asylum seekers with mental health needs (legal issues)		
Leaflets and how to use them		

### Evaluation of AMP training*plus*

We undertook a process evaluation to illuminate the process of initiating and delivering the training, and to identify where and why we succeeded against our aims and what lessons we should learn from the delivery of AMP training*plus*. After the training was completed, we sought to arrange interviews with practice members. Our sample consisted of everyone who was had attended at least one training session and was willing to speak to us. We conducted semi-structured interviews with individuals in 6 out of the 7 practices that had consented to and participated in training. Thirteen staff agreed to one-to-one interviews, while two practices preferred to give feedback in focus groups consisting of six participants in one and eight participants in the other. In total we received feedback from twenty seven participants. Each respondent gave informed consent for digital-recording of the interview or focus group and use of resulting data. We used topic guides to direct the flow of interviews and focus groups, and analysis was conducted to saturation by authors individually (CCG, HB, DH, SB, KG and MK) then agreed through discussion, using a framework analysis approach [32]. Analysis proceeded iteratively, and subsequent interviews were modified on the basis of findings from earlier ones. Data was initially organized using MAXQDA [33].

## Results

We delivered training in 7 out of 8 practices approached (See Table 2).

**Table 2:** Training sessions and staff, by practice

**Practice**	**Training sessions**	**Attending first session**	**Attending final session**
A	2	GPs (briefly), practice nurses, health practitioner, receptionists, administrative staff (19 in total)^1^	Practice manager, administrative staff (11 in total)
B	5	GPs (4), nurses (2), receptionist (1)	Administrative staff (3)
C	1	GPs (7)	(one session)
D	6	GPs (2), practice manager, administrative staff (5)	GP, practice manager, practice nurse, medical students (2)
E	1	GPs (5), nurses (1) receptionist & administrative staff (7), practice manager	(one session)
F	5	GPs (2), practice manager	GPs (3), practice manager
G	7	GPs (5), practice manager, practice nurse, Receptionists (8)	GPs (3), practice manager. Practice Nurse, Counsellor

### Reflections on the training process

We present results to illuminate the evaluation of the three interlinking strands of AMP training*plus.* Each illustrative data extract is identified by the source and an identifier.

### Knowledge transfer

Between 1 hour and 3 hours per practice was spent negotiating and organizing training. The aim was to make the training as flexible and accessible as possible in order to encourage practices to participate.

“*I think you were very supportive of our times and… very very flexible and accommodated us very well.”(*GP, practice D)

“ …*you did take on board our um expressed wishes or desires as to the type of thing we wanted to focus on.”* (PM, Practice D)

Reception staff reported that they found the training to be valuable, particularly the opportunity to learn about mental health issues and improve their understanding of patients they came into contact with across the desk.

“I just think it made you realise how many people out there have actually got this issue ........like with the older people and that you don't realise how many of the older people have that problem. It’s surprising but again, because it’s different races and things like that, that’s where the problems are and they keep it in don’t they?” (Receptionist Practice G)

GPs also suggested that including the receptionists in the training had had a positive impact on the practice:

*“Generally it was because it was for the patient, the reception staff as well because they have got a better understanding of how to deal with patients who are getting aggressive before… they think there might be something else going on. So rather than being just abrupt with they try to listen to the patients now more.”* (GP, Practice D)

Some reception staff suggested that the training might have impacted on how they would deal with patients in the future.

*“If you see someone who’s anxious and depressed you don’t go in with all guns blazing now …: like no, you can’t, it’s like right how can I help … I’ll try and do my best to help as long as you’re polite with me I’ll be polite with you.”* (Receptionist, Practice D)

From Table 2 it can be seen that the participation in training sessions varied between practices. Three practices participated in just one or two sessions whereas four practices agreed to five or more.

### Systems review

We asked respondents to reflect on changes in reception area and appointment-booking systems, and the role played by receptionists in mediation between GPs and patients.

There was some evidence of changes in systems within practices, which occurred after involvement in AMP, but not necessarily directly because of AMP:

I: I remember the first session I was at…..there was some talk about as well maybe changing the registration of new patients and getting N [health care assistant] more involved

PM: *Well that did go ahead…..so that was something, but that was, I think that was something, we probably would have gone to anyway, but it was still highlighted, if you know what I mean, because I know you talked to the girls about bits and pieces as well didn’t you*? (PM Practice F)

I: Did anything change after [AMP]?

R: *The appointment system changed and the phone system is improved now. It takes the pressure off us [receptionists]. There are far fewer complaints about the system now.* (Receptionist Practice E)

There was a feeling that involvement in training had created a space for members of the practice to come together, have discussions and put into action (sometimes long held) plans for improvement. Also, there seemed to be increased awareness of mental health issues generally,

I wonder what has changed in the practice over the last couple of years in regard to mental health?

PM: *Erm, it seems to be discussed an awful lot more at our Tuesday meetings* (PM Practice F)

The opportunity to work together seemed to have enabled a dialogue that had not been possible before, and led to better understanding of each other’s experiences in the practice:

*“Oh yes, yes, yes, we enjoyed, we loved that [patient journey session] because it was very interactive, very, very interactive. And they gave the staff the opportunity to because we don’t all see what’s happen outside, patient come in often extremely nice to us, but when you go out and the staff there ‘oh, he was horrible to me’. So we don’t know what happens outside so we learnt about their side of stories as well.”* (GP, Practice D)

### Active linking

We invited respondents to reflect on whether the training had raised awareness of community organisations and other resources as referral options for people with mental health problems. We found from our initial ethnographic study a surprising lack of awareness of community resources even when they were located in close proximity to the practice (i.e. in the same street in one case) There was evidence that we did raise awareness about the community organisations, although it was not clear from these interviews how much this translated into changed behaviours:

*“They were very useful - it made the practice team aware of existence of groups and services that we never thought they existed around us…..Not very far from us as well…..Also it gave us a clear way of tapping into these services whenever we need them so that is a great benefit.....I haven’t personally referred because I probably haven’t come across a patient…..The fact that we are aware of their presence and we have got folders and leaflets to tell us who they are where they are……Telephone for contact and email addresses I think it’s a great result*.” (GP, Practice B)

“*… I think we all became more aware of services that are in the community that we didn’t know about even though they were probably on the doorstep almost.”* (Practice nurse Practice B)

## Discussion

AMP training*plus* was delivered to a greater or lesser degree to seven practices, but it is difficult to explain why some practices participated in more training sessions than others. We can tentatively say, however, that increased participation seemed to be due to the commitment of both the practice manager and at least one of the lead GPs. Practices which did participate in more than one session involved the whole practice including the clinical staff, practice manager and the administrative staff in the training, as we had intended.

In terms of achieving our four intended outcomes:

*Increased staff awareness, recognition and respect for diversity*: Staff at all levels within primary care teams that engaged with the training programme reported increasing awareness, recognition and respect for the needs of patients from under-served communities. We cannot be confident of the extent to which such reports were evidence of substantial changes in staff attitudes, or whether they may have been influenced by other factors, such as a wish to convince the AMP team that our efforts had been worthwhile.

*Change in consultation or encounter style and content*: We received reports of changes in encounter style and content particularly amongst receptionists. We are relying primarily on verbal evidence from practice respondents here, which may be subject to bias. The robustness of these findings would have been enhanced by follow-on ethnographic work with reception staff, or by before-and after assessment of the style and content of GP consultations with patients from under-served groups.

*Change in processes and systems*: Within several practices, there were indications that reflecting back our ethnographic findings [29] gave reception staff greater confidence in their role as mediator between GPs and under-served patients. There was also evidence of a system change in one practice (around triage), although it was not clear whether this was a result of, or coincidental with, engagement with the AMP training programme. It may be that the practice staff members were more confident after their training session to make suggestions for change to their GP employers. There was no evidence of any changes in the systems for the use of interpreters. As stated, efforts to collect data around referrals did not lead to useful data being collected.

We found evidence that the training programme had increased awareness of – and encouraged signposting to - relevant community organizations within the practice locality. This was particularly the case in the localities where the AMP community engagement intervention was taking place, but it also happened in the other two localities. However, we do not know whether or to what extent such signposting and patient referral actually occurred.

### Relevance to the published literature

It is clear from this study that, as the literature suggests [4-7], improving outcomes via GP training is not straightforward or easy to achieve. We have demonstrated, however, that involving the whole practice in training may be an unusual approach but it does have substantial benefits. In keeping with previous studies [13,16], it is crucial to understand what is already happening within the complex system of a practice so that training can tailored to individual need. The ethnographic style of investigation used to achieve this required intensive research but we feel it provided real insights which we were able to feed back to the practices. Similarly, in accordance with previous work [17] our ethnographic investigation into the local communities provided an understanding of the context of patients’ lives along with local needs and resources. This helped us to offer training that was genuinely relevant and endorses previous findings [21].

## Conclusion

### Strengths and weaknesses of the study

This study is among the first in the UK to propose Primary Care Quality Improvement in the context of a wider system change at locality level. We were able to engage with practices despite the many local and national pressures bearing upon them. Also, the finding that all levels of the practices team appreciated the benefits of involving receptionists in the training confirms the benefits of this approach.

Weaknesses of the study are that our engagement with some practices was limited and we were unable to collect objective, quantitative evidence of reported changes e.g. in referral behaviour. In addition, we were able to conduct one-to-one interviews with only 13 staff despite active attempts to arrange more. The most common explanations received were lack of available time for GPs and lack of cover available for receptionists working on the desks. After the training was completed it was difficult, despite numerous approaches, to engage further with individuals.

### Implications for clinical practice, policy and research

This study shows that it is possible to develop, offer and deliver a flexible package of training to a whole practice. The Practice has to be understood and approached as a complex organization, however, and the ethnography [29] we carried out prior to beginning the training was a great help in forging links, understanding how the practice worked, and agreeing training priorities.

In retrospect, there was a tension between our aim as researchers to be flexible with regards to the content and delivery of the training, and our wish to deliver a programme of training, covering a number of areas. If practices did not identify further learning needs, we were not able to suggest more training. This perceived flexibility also worked against us as we attempted to gain access to practice staff for interview. If we were to conduct this work in further practices, we would perhaps be less flexible, in order to conduct similar amounts of training in each practice and stress the importance of the interviews and focus groups as part of the agreement for practices participating.

The importance of involving receptionists and including information about community agencies is clear, as is the use of the AMP model as a template for further training design and research in this field. There is a need for future studies to include substantive quantitative elements in evaluation, to demonstrate changes in, for example, health care use and referral patterns.

### Ethical approval

Ethical approval was granted by Northwest 6 Research Ethics Committee — GM South (REC reference: 09/H1004/67).

## Competing interests

The authors declare that they have no competing interests.

## Authors’ contributions

CCG contributed to development of the study, data analysis and writing the paper. HB contributed to data collection and analysis, and writing the paper. DH contributed to data collection and analysis, and writing the paper. LG contributed to development of the study and writing the paper. SB contributed to data collection and analysis and writing the paper. KG contributed to data collection and analysis, and writing the paper. WW contributed to writing the paper. MG contributed to writing the paper. MK contributed to data collection and analysis,and writing the paper. CD contributed to development of the study and writing the paper. All authors read and approved the final manuscript.

## Pre-publication history

The pre-publication history for this paper can be accessed here:

http://www.biomedcentral.com/1471-2296/15/68/prepub

## Prior presentation

Material in this paper has previously been presented as ‘Developing Primary Care’ at the World Psychiatric Association, Granada 2012, and ‘Improving Access to Primary Mental Health Care in Underserved Populations’ at the World Psychiatric Association, Bucharest 2013.
